# Time-aligned segmental strain as a diagnostic marker for obstructive coronary artery disease in patients with chronic chest pain

**DOI:** 10.1186/s12947-026-00388-0

**Published:** 2026-10-19

**Authors:** Assami Rösner, Hatice Akay Caglayan, Mehran Jazbani, Semra Øztemel Sari, Pablo Marti Castellote, Didrik Kjønås, Sandro Queirós

**Affiliations:** 1 Department of Cardiology, Division of Cardiothoracic and Respiratory Medicine, University Hospital of North Norway, Tromsø, 9019 Norway; 2 Department of Clinical Medicine (IKM), UiT The Arctic University of Norway, Tromsø, 9019 Norway; 3 Department of Cardiology, Stavanger University Hospital, Stavanger, Norway; 4 Department of Information and Communication Technologies (DTIC), Universitat Pompeu Fabra (UPF), Barcelona, Spain; 5 Department of Gastrointestinal Surgery, University Hospital of North Norway, Tromsø, Norway; 6 Life and Health Sciences Research Institute (ICVS), School of Medicine, University of Minho, Braga, Portugal; 7 2Ai - Applied Artificial Intelligence Laboratory, School of Technology, Polytechnic University of Cávado and Ave (IPCA), Barcelos, Portugal

**Keywords:** Chronic coronary artery disease, Speckle tracking analysis, Segmental strain, Time-aligned strain, Time-aligned Strain rate

## Abstract

**Background:**

Myocardial strain curves provide valuable insights into coronary artery disease (CAD) beyond conventional peak strain measurements. This study aimed to evaluate the diagnostic utility of time-aligned segmental strain and strain rate (S/SR) curves to identify obstructive CAD in consecutive chest pain patients.

**Methods:**

A total of 510 chest-pain patients referred to coronary computed tomography angiography (CCTA) were included. Segmental strain curves were time-aligned using aortic valve closure (AVC) and cardiac cycle length as reference points. Average S/SR curves were generated for 18 myocardial segments, establishing reference curves from a group of 314 chronic chest-pain patients without CAD (No-CAD). Deviations were defined based on thresholds of peak S/SR or average S/SR curve deviation over systole and the first half of diastole.

**Results:**

Significant differences were observed between groups, particularly between the revascularized MI, CABG, or 3-vessel CAD groups compared to the No-CAD group (p<0.05). ROC curve analysis demonstrated excellent diagnostic performance for CABG patients (AUC up to 0.933), while the detection rate for PCI was limited (AUC < 0.7). Global longitudinal strain rate in early diastole (GLSR E) and segmental peak SR E showed the highest diagnostic accuracy. These findings confirm that time-aligned strain analysis improves the detection of significant CAD but remains limited for identifying patients with mild disease.

**Conclusion:**

Time-aligned segmental strain analysis improved detection of high-grade disease requiring CABG but could not distinguish single-vessel from non-obstructive CAD. Pathological strain patterns became significant only in advanced CAD, indicating that segmental resting strain analysis has clinical utility primarily for identifying advanced CAD.

**Graphical Abstract:**

**Figure:**
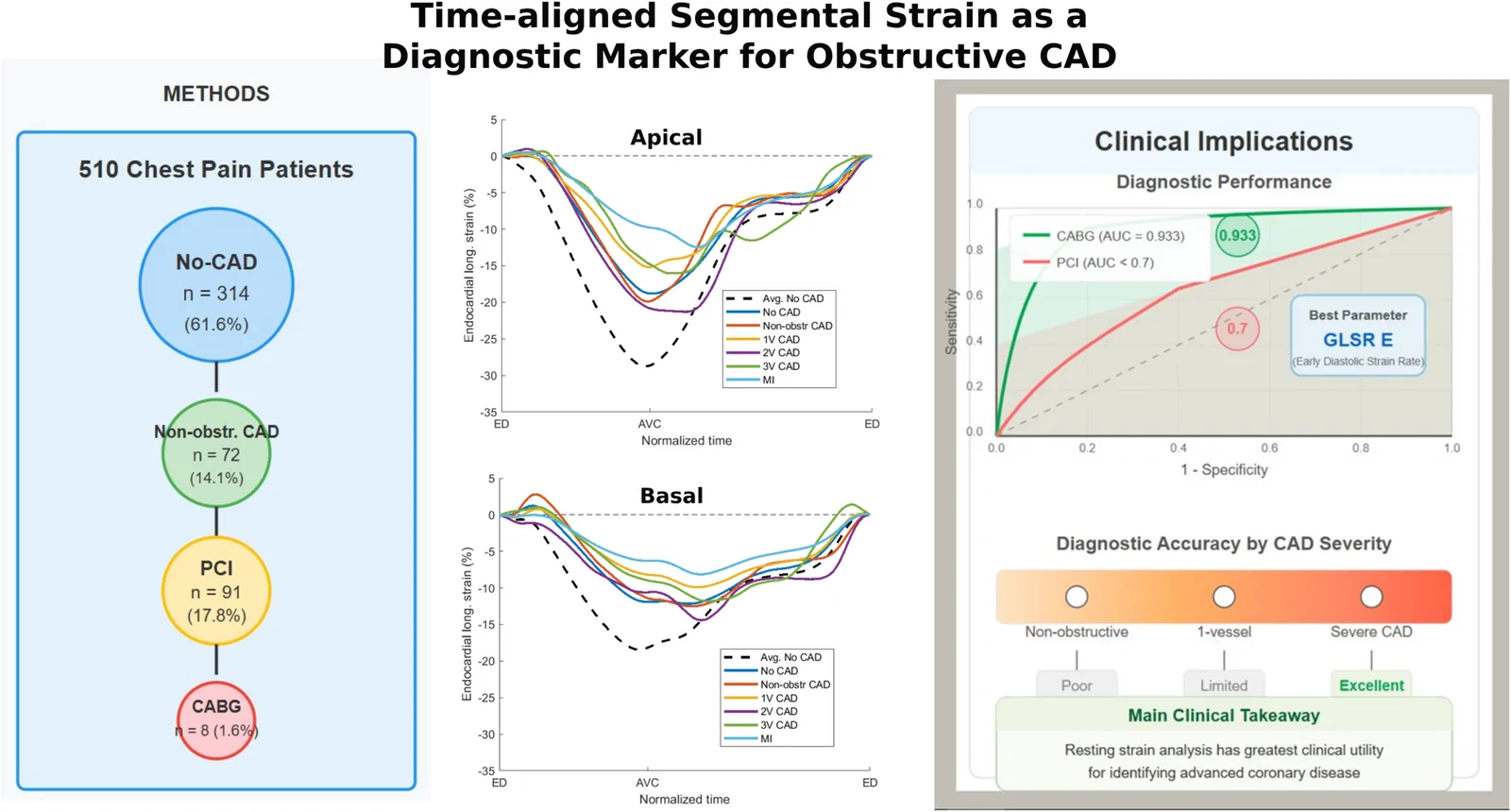

**Supplementary Information:**

The online version contains supplementary material available at https://doi.org/10.1186/s12947-026-00388-0.

## Key phrases

- Time-aligned segmental strain detects severe coronary artery disease with excellent accuracy (AUC 0.933).
- Early diastolic strain rate (GLSR E) is the most powerful diagnostic parameter for CAD detection.
- Cannot reliably distinguish single-vessel from non-obstructive coronary artery disease.
- Pathological strain patterns become significant only in advanced coronary disease.
- Resting echocardiography has greatest utility for identifying high-risk CAD patients requiring CABG.

## Key takeaways

- Early diastolic strain rate (GLSR E and segmental SR E) demonstrated superior diagnostic performance for detecting significant coronary artery disease compared to other strain parameters.
- Time-aligned segmental strain analysis excellently detects severe CAD requiring CABG but cannot reliably distinguish single-vessel disease from non-obstructive CAD.
- Pathological strain patterns become significant only in advanced coronary artery disease, showing characteristic features of delayed contraction, reduced systolic shortening, and post-systolic strain.
- Resting echocardiographic strain analysis has greater clinical utility for identifying high-risk patients with advanced coronary disease rather than those with mild or single-vessel disease.

## Introduction

Coronary artery disease (CAD) remains a leading cause of morbidity and mortality worldwide, presenting a substantial healthcare challenge that emphasizes the need for detecting patients at risk [1]. However, only about 15% of all patients presenting with chronic chest pain to primary healthcare facilities are diagnosed with obstructive CAD [2]. Computed tomography coronary angiography (CTCA) is a highly sensitive tool for identifying patients with CAD [3]; however, its radiation exposure, availability, and costs necessitate careful pre-test patient selection. Resting echocardiography may offer greater potential as a screening tool, as it can be applied repeatedly without side effects and lower costs. Recent advancements in imaging techniques, specifically two-dimensional speckle tracking echocardiography (2DSTE), allow for detailed myocardial strain and strain rate measurements, providing insights into myocardial deformation that may enhance CAD detection sensitivity and specificity [4, 5].

Strain imaging, particularly global longitudinal strain (GLS), has shown promise in identifying subclinical myocardial dysfunction in various CAD populations [6, 7]. GLS can detect functional impairments even in patients with preserved left ventricular ejection fraction (LVEF), positioning it as a valuable tool in risk stratification for CAD patients with stable angina [8]. Furthermore, studies have demonstrated that abnormal segmental strain patterns, especially post-systolic shortening (PSS), correlate with increased CAD severity, highlighting the clinical relevance of analyzing strain curves over simple peak strain values [9].

In patients with chronic CAD, segmental strain analysis reveals deformation patterns across different ischemic pathologies, enabling a comprehensive assessment of myocardial function. This detailed analysis has been shown to improve diagnostic accuracy for CAD, particularly when traditional methods might miss subtle regional dysfunction [10, 11]. Additionally, strain curve deviations, including delayed shortening and PSS, have been identified as markers of myocardial ischemia, offering a potential non-invasive approach for identifying high-risk patients [12].

The RECCAD study (can Resting Echocardiography identify patients with Chronic Coronary Artery Disease? ) aims to expand upon these findings by examining segmental strain and strain rate curves in a stable chest pain population referred for coronary computed tomography angiography (CCTA) [13]. By analyzing segmental strain curve deviations from reference segments and comparing groups with various levels of coronary obstruction, this study seeks to define strain curve patterns in different degrees of CAD, including patients who underwent revascularization after acute myocardial infarction.

Incorporating both quantitative strain metrics and degrees of curve deviation from segmental reference curves, the study hypothesizes that segmental strain analysis will reveal diagnostic markers indicative of CAD, potentially aiding in the early identification of myocardial ischemia. This approach aligns with a growing body of literature advocating for the role of strain imaging in CAD diagnostics, where it has shown utility in differentiating ischemic from non-ischemic cardiomyopathy and predicting adverse cardiac events [14–17].

This study will present findings on the diagnostic utility of segmental strain curves vs. peak values in CAD patients, addressing the sensitivity and specificity of identifying patients with varying degrees of chronic CAD. The study also aims to characterize typical strain-curve formations associated with different degrees of CAD, including patterns in revascularized patients, post-acute myocardial infarction.

## Methods

### Study population

This prospective observational study, conducted from 2016 to 2021, included a cohort of 510 chest pain patients and 102 myocardial infarction (MI) patients recruited from the Cardiology Department at the University Hospital of North Norway, Tromsø.

Patients in the chest pain group were referred for CCTA based on an initial assessment by cardiologists that included chest pain history, exercise electrocardiography (ECG), family history, and associated risk factors. The RECCAD study cohort has been presented in an earlier publication [13]. Exclusion criteria for this group included atrial fibrillation (AF), left bundle branch block (LBBB), previous CAD, and any structural heart disease. Based on findings from CCTA and coronary angiography (CAG), patients were categorized into one of five chronic chest-pain groups based on the degree of coronary obstructions, namely No-CAD (without coronary plaques in CCTA), non-obstructive CAD, 1- ,2-, and 3-vessel disease, or into one of four groups based on treatment, namely No-CAD-, non-obstructive CAD-, PCI-, and CABG-group.

For the MI group, patients were included if they had experienced an acute ST-elevation myocardial infarction (STEMI) or Non-ST-elevation myocardial infarction (NSTEMI) within 1–3 days post-percutaneous coronary intervention (PCI). Exclusion criteria for the MI group included a prior history of MI or non-revascularization due to referral for coronary artery bypass grafting (CABG).

### Clinical characteristics

All participants completed a questionnaire covering symptoms and risk factors, and relevant clinical data such as lipid profile, creatinine, HbA1c, and fasting glucose levels were obtained from electronic medical records. Prior to echocardiographic examination, blood pressure, height, weight, and body mass index (BMI) were measured, and a 12-lead ECG was recorded. Diabetes mellitus (DM) was defined as a history of DM treatment, fasting glucose > 126 mg/dl (7.0 mmol/L), or HbA1c > 6.5% (48 mmol/mol). Hypertension (HT) was defined as either treatment for HT or blood pressure > 140/90 mmHg.

### Echocardiography, strain, and strain rate analysis

Chest pain patients underwent echocardiography on the same day as CCTA, and MI patients were examined 1–3 days after PCI. All echocardiograms were performed in the left lateral decubitus position using a Vivid E9 or E95 ultrasound machine (GE, Horton, Norway) with a 5 − 1 MHz transducer. Standard two-dimensional (2D) grayscale images were acquired from parasternal long and short axes, as well as apical two-, three-, and four-chamber views. Doppler flow data across the mitral valve, left ventricular outflow tract (LVOT), and aortic valve were obtained from apical views. Peak R of the QRS complex was marked as the beginning of the cardiac cycle to standardize segmental peak strain calculations. Three consecutive cardiac cycles were captured at rest in each apical view, with frame rates set above 45 frames per second. All images were stored digitally in cine-loop format for strain analysis.

Strain analyses of 2D images were conducted offline using the Q-analyses function in EchoPac software (GE), analyzing one of three cycles. Aortic valve closure (AVC) was identified as end-systole, with pulsed wave Doppler of LVOT used to confirm aortic valve timing. The endocardial border was manually traced at end-systole for strain analysis. Strain and strain rate (S/SR) curves were automatically generated for all six segments in each view, and visual inspection of curves was used to identify and discard artifacts. The identification of strain-curve artifacts by unphysiological deformation features has been described in an earlier publication of the study group [18].

### Definition of deviating segmental curves

**Fig. 1:**
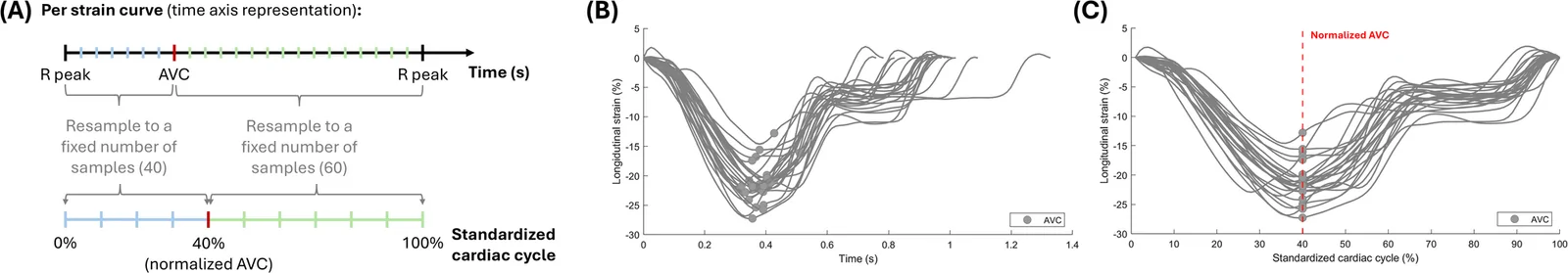
Time alignment and standardization of segmental longitudinal strain curves. **A** Schematic illustration of the resampling procedure used to standardize each segmental strain curve within the cardiac cycle. The interval from the R peak to aortic valve closure (AVC) was resampled to a fixed number of points, and the interval from AVC to the next R peak was resampled separately, yielding a standardized cardiac cycle in which AVC was fixed at 40% of cycle length. **B** Example of raw segmental longitudinal strain curves plotted against absolute time, showing inter-individual variation in cycle length and timing of AVC. **C** The same curves after time normalization, demonstrating alignment of AVC and improved comparability of segmental deformation patterns across subjects

S/SR curves for all participants were time-aligned to a standardized cardiac cycle length and time to AVC, as illustrated in Fig. 1. Specifically, each curve was resampled to a fixed length (100 points per cardiac cycle), with AVC aligned to the same relative position (40% of the cycle) by separately interpolating the systolic and diastolic phases. Segmental reference curves were generated by averaging the time-aligned curves from 314 individuals in the chest pain cohort (No-CAD group) who had either no evidence of coronary artery disease or stenoses < 30%, no valvular pathology, myocardial disease, or bundle brunch block (QRS > 120 ms). These reference curves were defined for each of the 18 myocardial segments. For each study participant, deviations from the corresponding reference segmental curves were quantified in two ways: as the difference between the participant’s segmental peak systolic and diastolic S/SR values and the respective reference peak values, and as the average difference between the participant’s segmental curve and the corresponding reference curve over systole for S/SR and the first third of diastole for strain rate. For visualization purposes, pathological thresholds were defined as the 5th percentile lower bound of segmental deviations observed in the No-CAD group. Global longitudinal strain (GLS), systolic strain rate (GLSR-S), and early diastolic strain rate (GLSR-E) were also included in the analysis for their potential diagnostic value. In addition, we defined time-aligned reference average S/SR curves in order to highlight possible differences between use of segmental or average S/SR references.

### Invasive coronary angiography

Patients with positive or inconclusive CCTA results and all MI patients underwent invasive CAG via either radial or femoral access. Stenosis was considered significant if there was a ≥ 70% reduction in the lumen of the right coronary artery (RCA), left anterior descending artery (LAD), or circumflex artery (CX), or a ≥ 50% reduction in the left main coronary artery (LMCA). Only significant stenoses were classified as 1-, 2- or 3- vessel disease and grouped as such. Patients with main stem stenosis and at least one-vessel disease were classified as 3-vessel disease.

Patients with significant stenosis were revascularized as needed (PCI group), and those requiring surgical intervention were referred to CABG. Patients with coronary stenoses 30–70% at CAG were classified as non-obstructive CAD while the No-CAD group included none to < 30% stenoses.

### Statistical analysis

Statistical analyses were performed using SPSS software version 29 (SPSS Corp., Armonk, NY: IBM Corp, USA). Continuous variables are reported as mean ± standard deviation, while categorical data are presented as absolute numbers or percentages. Comparisons of patient characteristics among the No-CAD, Non-obstructive CAD, 1-vessel, 2-vessel and 3-vessel disease, or PCI, CABG, and MI groups were conducted using one-way analysis of variance (ANOVA) with Bonferroni post-hoc tests for continuous variables and chi-square tests for categorical variables. ANOVA with Bonferroni post-hoc tests was also used to compare S/SR curve deviations across groups, as well as the percentage of pathological segments across deviation groups. Receiver operating characteristic (ROC) curve analysis was conducted to determine the accuracy of detecting patients requiring either PCI or CABG treatment compared to participants with No-CAD or non-obstructive CAD in the chest pain population. In all analyses, p-value < 0.05 was considered statistically significant.

### Reproducibility

Inter- and intra-observer variability for strain-curve analysis of the study has been published earlier [13].

## Results

Patient characteristics across diagnostic and treatment groups are presented in Table 1, with additional results from this cohort previously published [13]. The study included 88 patients who underwent revascularization for acute myocardial infarction and received echocardiography 1–3 days post-procedure. From the initial 510 patients assessed for chronic chest pain, 485 were included in the final analysis, while 25 were excluded due to pre-existing cardiac conditions, such as valvular heart disease, bundle branch block, previous myocardial infarctions, cardiomyopathies, myocarditis and atrial fibrillation. Demographics analysis revealed a higher proportion of males in CAD groups, including both stenotic and non-stenotic CAD, with these patients also being significantly older than those without CAD. The CABG group, though smaller in size, exhibited significantly higher body weight and systolic blood pressure compared to other groups. Patients with CAD showed higher prevalence of hypertension and diabetes, along with elevated creatinine levels. Troponin T levels were significantly higher in the PCI group compared to the No-CAD group.

**Table 1:** Patient characteristics of the chest-pain population

	No-CAD		Non-obstr.CAD		PCI		CABG		p-value
n	314		72		91		8		
	Mean/n	STD/%	Mean/n	STD/%	Mean/n	STD/%	Mean/n	STD/%	
Female	173	52	25	35*	23	25*	2	25	<0.001
Male	160	48	46	65*	68	75*	6	75	
Age	57.8	±10.8	62.4*	±9.6	62.7*	±9.1	61.3	±10.5	<0.001
Height (cm)	170	±10	172	±8.8	174*	±8.5	170	±9.6	0.008
Weight (kg)	78.0	±15.6	79.8	±12.5	83.0*	±13.1	88.2*	±13.0	0.012
BMI (kg/m2)	26.4	±3.4	26.3	±3.0	27.3	±3.0	31.1	±5.0	0.003
CAD Family hx	200	60	52	73	57	63	6	75	0.229
HR (min^-1^)	66.0	±11.8	68.6	±11.8	65.7	±11.4	73.8	±9.6	0.096
QRS duration (ms)	96.7	±13.6	96.4	±10.9	100.4	±14.9	100.3	±11.1	0.120
BP sys (mmHg)	137	±18	143	±19	143	±20	155*	±22	0.001
BP dia (mmHg)	87	±11	87	±9	86	±11	93	±9	0.421
Diabetes	17	5	7	10*	12	13*	1	13	0.035
Hypertension	93	28	31	44*	37	41	3	38	0.014
COPD	20	6	5	7	9	10	1	13	0.585
Creatinin (µmol/L)	73.3	±14.1	75.3	±17.5	79.1*	±18.3	76.5	±11.7	0.024
Smoked daily	50	15	13	18	20	22	3	38	0.206
HS Trop T (ng/L)	6.7	2.3	7.5	3.9	10.4*	10.5	6.0	0.0	<0.001

*CAD *Coronary artery disease, *PCI* Percutaneous coronary intervention, *CABG* Coronary artery bypass graft, *BMI* Body mass index, *HR* Heart rate, *BP B*lood pressure, *COPD* Chronic obstructive pulmonary disease, *HS Trop T* High sensitive Troponin T

*p<0.05 comparison towards No-CAD group

### Segmental strain analyses

We evaluated segmental curve deviations using both peak values and deviations over specific portions of S/SR curves which are shown in Table 2, including systolic and early diastolic global longitudinal (GL) S/SR.

**Table 2:** Segmental and global parameters in different states of coronary artery disease

	No-CAD	Non obstructive CAD	1 V Disease	2 V Disease	3 V Disease	PCI	CABG	Revasc MI
*n*	314	72	74	18	7	91	8	88
Segm Curve dev systole (%)	-0.03 ± 1.61	0.14 ± 1.77	-0.14 ± 1.85	0.28 ± 1.91	-2.16 ± 1.83	-0.03 ± 1.84	-2.30 ± 1.72*	-3.20 ± 3.08*
Segm Curve dev E-wave (s^− 1^)	-0.00 ± 0.14	-0.01 ± 0.13	-0.04 ± 0.14	-0.06 ± 0.14	-0.17 ± 0.15	-0.05 ± 0.14	-0.17 ± 0.14*	-0.21 ± 0.19*
Segm PL Strain dev (%)	-0.03 ± 2.61	-0.07 ± 3.11	-0.96 ± 3–03	-0.02 ± 2.95	-3.56 ± 3.46	-0.74 ± 3.00	-3.8 ± 3.26*	-6.24 ± 5.63*
Segm PL SR S dev (s^− 1^)	-0.01 ± 0.17	0.02 ± 0.16	-0.04 ± 0.19	-0.02 ± 0.20	-0.04 ± 0.14	-0.03 ± 0.19	-0.07 ± 0.17*	-0.18 ± 0.27*
Segm PL SR E dev (s^− 1^)	-0.01 ± 0.29	-0.05 ± 0.32	-0.14 ± 0.28*	-0.20 ± 0.27	-0.50 ± 0.08*	-0.15 ± 0.28*	-0.47 ± 0.09*	-0.47 ± 0.36*
GLS (%)	-19.0 ± 2.4	-18.7 ± 2.8	-18.1 ± 2.8	-18.4 ± 3.2	-14.7 ± 2.4*	-18.2 ± 2.9	-14.8 ± 2.3*	-14.4 ± 4.1*
GL SR S (s^− 1^)	-0.98 ± 0.15	-0.98 ± 0.16	-0.95 ± 0.18	-0.96 ± 0.18	-0.86 ± 0.13	-0.96 ± 0.17	-0.85 ± 0.14	-0.82 ± 0.25*
GL SR E (s^− 1^)	1.20 ± 0.31	1.14 ± 0.34	1.07 ± 0.28*	1.00 ± 0.33	0.75 ± 0.14*	1.06 ± 0.29*	0.76 ± 0.15*	0.89 ± 0.31*

*dev* deviation, *Segm* Segmental, *GLS* Global longitudinal strain, *GL SR S* Global longitudinal strain rate in systole, *GL SR E* Global longitudinal SR early diastole, *1,2,3, V* 1,2,3, Vessel, *PCI* Percutaneous coronary intervention, *CABG* Coronary artery bypass graft, *Revasc MI* Revascularized myocardial infarction

*significant difference towards the No-CAD group

As expected, patients in the revascularized MI group exhibited substantial deviations across all parameters tested, with similar patterns observed in those from the CABG and 3-vessel (3 V) CAD groups. Despite the relatively small size of the CABG group, significant differences were observed compared to the No-CAD patients. Notably, only segmental peak SR E and GLSR E deviations showed significant differences between PCI or 1-vessel (1 V) CAD groups and the No-CAD group.

**Fig. 2:**
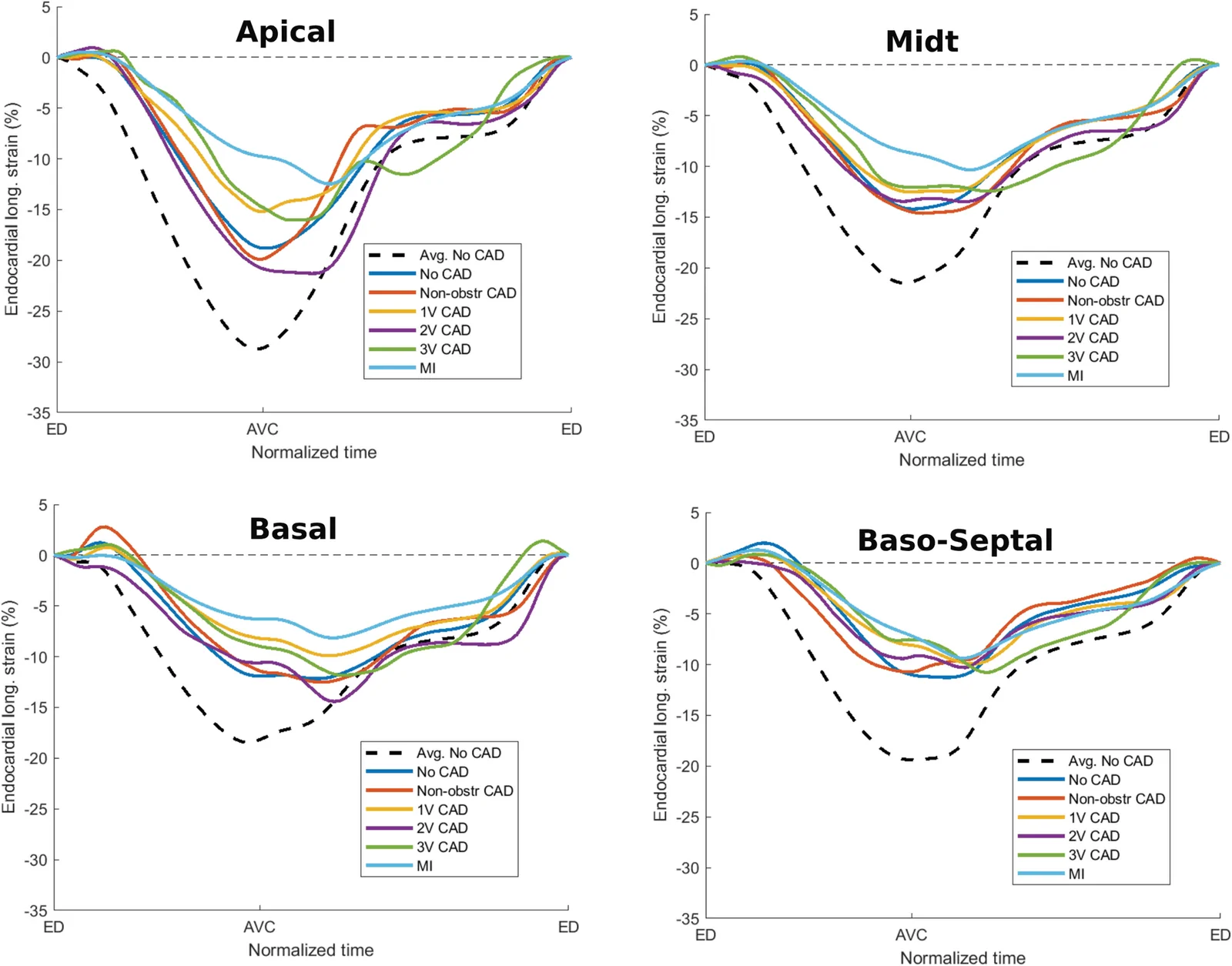
Average time-aligned segmental longitudinal strain curves in apical, mid-ventricular, basal, and baso-septal regions across CAD groups and revascularized MI. The dashed black line shows the No-CAD reference curve; colored lines show average pathological curves with systolic deviation below the 5th percentile of the segment-specific reference

**Fig. 3:**
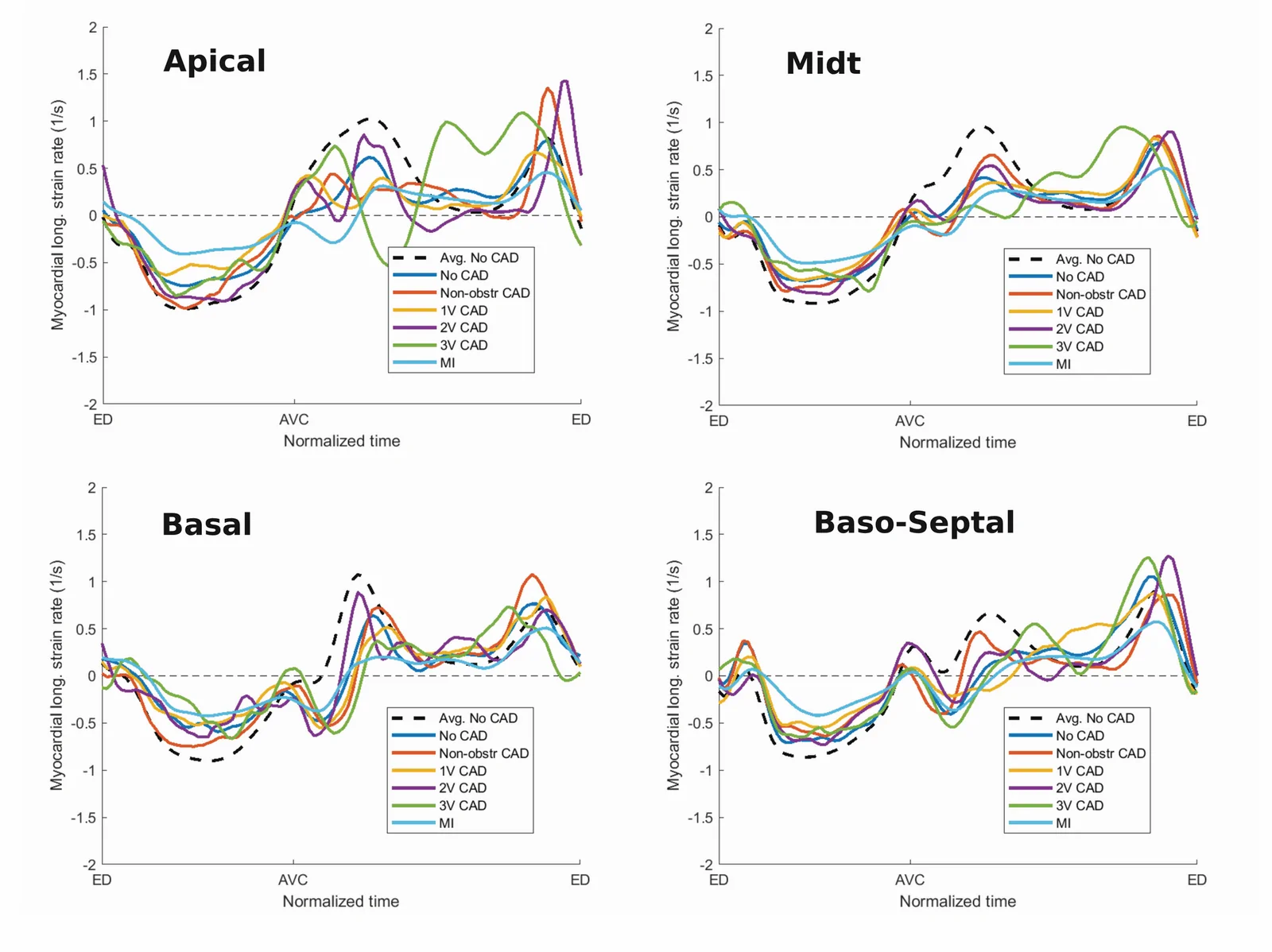
Average time-aligned segmental longitudinal strain-rate curves in apical, mid-ventricular, basal, and baso-septal regions across CAD groups and revascularized MI. The dashed black line shows the No-CAD reference curve; colored lines show average pathological curves with early diastolic deviation below the 5th percentile of the segment-specific reference

Figures 2 and 3 depict the average time-aligned segmental strain and strain rate curves, respectively, across different degrees of CAD and revascularized MI. For both figures, curves were derived by averaging segmental curves classified as pathological—defined as those exceeding the 5th percentile of deviation from the corresponding segmental reference curve established in the No-CAD group. The corresponding average reference curves from the No-CAD group are included for comparison. Supplemental Figures S1 and S2 show average segmental strain and strain rate curves, respectively, within the No-CAD group that fall within specific percentile ranges of deviation from the segmental reference curves (specifically, the 10–25th, 5–10th, and below 5th percentiles). Deviations were calculated over systole for strain (Figure S1) and over the first third of diastole for strain rate (Figure S2), as defined in the Methods. This stratification supports visual interpretation of what constitutes a normal versus pathological strain-curve.

Early diastolic strain rate curves showed the most significant deviations between groups based on CAD severity. Specific features such as peak early diastolic strain rate and double peaks during early diastole may reveal additional characteristics that were not incorporated into the present data analysis.

### Comparison of segmental vs. global S/SR approaches

The supplemental Table S1 compares the detection rates of pathological segments across all grades of CAD in four segmental groups, contrasting two different reference approaches: one using segment-specific reference curves (time-aligned for each of 18 individual segments) versus another using a single global average curve (applied across all segments) to identify significant deviations (> 95% deviation toward lower values). Notably, apical segment pathologies were frequently missed when using SR E without definition of segmental references—28.3% of MI patients showed pathologies with segmental reference versus only 19.5% when using global normal values as reference. Mid-ventricular segments showed the least dependency on segmental reference values, while basolateral and basoseptal segments with reduced S/SR were disproportionately classified as functionally abnormal in the global cut-off group. Despite these regional differences, the overall number of pathological segments remained similar between segmentally corrected and non-corrected cut-off values. Nevertheless, segmental-based definition of pathological deviation appeared to improve diagnostic accuracy, particularly in identifying abnormal apical segments. The necessity for segmental definition of reference values was most evident for SR E parameters.

### Diagnostic performance

**Fig. 4:**
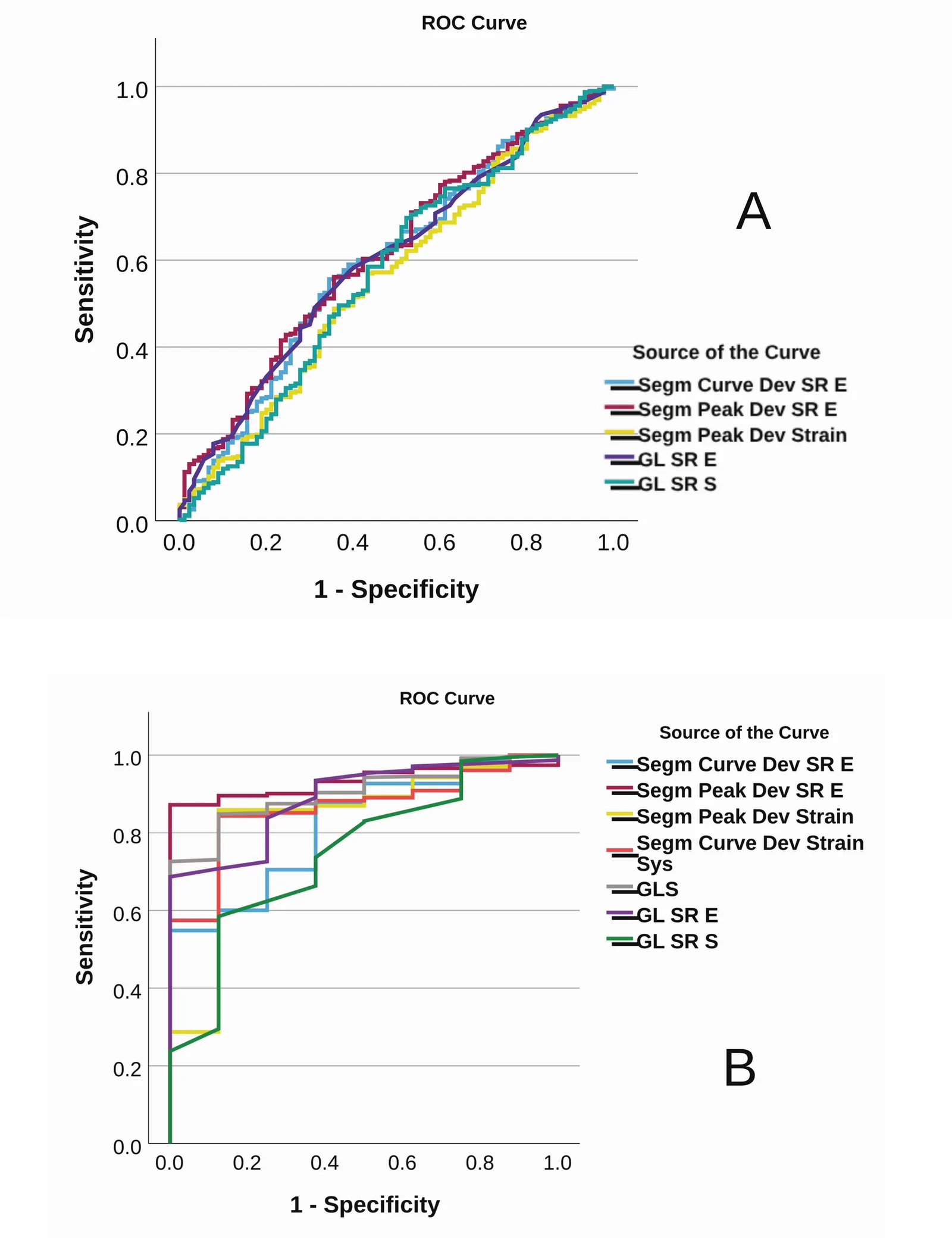
Receiver operating characteristic curves for strain and strain-rate parameters identifying patients requiring revascularization: (**A**) PCI versus No-CAD/non-obstructive CAD and (**B**) CABG versus No-CAD/non-obstructive CAD. Diagnostic performance was limited for PCI and markedly better for CABG, especially for early diastolic strain-rate parameters

We conducted ROC curve analysis for all parameters to evaluate their ability to distinguish PCI (Fig. 4A) and CABG patients (Fig. 4B) from all chest-pain patient without indication for revascularization (No-CAD and non-obstructive CAD). Table 3 presents the respective area under the curve (AUC) values, along with cut-off values, sensitivity, and specificity for parameters with AUC > 0.8.

**Table 3:** ROC curve analysis comparing No-CAD and non-obstructive CAD against PCI (excluding CABG) and CABG (excluding PCI)

	PCI		CABG				
	AUC (CI)	*p*-value	AUC (CI)	*p*-value	Cut-off	Sensitivity (%)	Specificity (%)
Segm Curve dev strain systole (%)	0.505 (0.437–0.573)	0.891	**0.864 (0.777–0.951)**	**< 0.0001**	-1.5	86	88
Segm Curve dev E-wave (s^− 1^)	**0.601 (0.535–0.666)**	**0.003**	**0.820 (0.703–0.938)**	**< 0.0001**	-0.30	84	100
Segm PL Strain dev (%)	**0.562 (0.505–0.643)**	**0.034**	**0.835 (0.686–0.985)**	**< 0.0001**	-2.8	86	88
Segm PL SR S dev (s^− 1^)	**0.574 (0.505–0.643)**	**0.035**	0.625 (0.418–0.832)	0.235			
Segm PL SR E dev (s^− 1^)	**0.618 (0.555–0.682)**	**< 0.0001**	**0.933 (0.900-0.967)**	**< 0.0001**	-0.34	87	100
GLS (%)	0.565 (0.499–0.630)	0.053	**0.904 (0.843–0.965)**	**< 0.0001**	-16.5	85	88
GL SR S (s^− 1^)	0.534 (0.466–0.603)	0.322	0.750 (0.591–0.910)	0.081			
GL SR E (s^− 1^)	**0.605 (0.542–0.669)**	**0.001**	**0.889 (0.809–0.969)**	**< 0.0001**	0.97	71	87

*Segm* Segmental, *GLS* Global longitudinal strain, *PL* Peak longitudinal, *GL SR S* Global longitudinal strain rate in systole, *GL SR E* Global longitudinal SR early diastole, *CI* Confidence interval Cut-off values for sensitivity and specificity were generated for AUC > 0.80

Bold data represent significant results, as indicated by the *p*-value column

The diagnostic performance for detecting PCI patients was generally poor, with AUC values < 0.7, indicating limited ability to identify patients requiring PCI. In contrast, detection of CABG patients was excellent, with AUC values reaching 0.933. In both analyses, peak SR E deviation and GLSR E emerged as the best indicators among all tested parameters.

The supplemental Table S2 demonstrates how various strain parameters distinguish between patients without CAD and with non-obstructive CAD versus those with revascularized infarctions. The AUC values were notably lower compared to the ROC curve analyses in the CABG group (< 0.8), suggesting that some patients achieve normal global and segmental function due to revascularization. Notably, segmental SR E showed clear superiority over GLSR E, while segmental strain performed similarly to GLS.

## Discussion

In this study, we introduced a novel methodology that defines time-aligned segmental strain and strain rate curves to a standard reference curve for each myocardial segment, then classified individual curves based on their deviation from either peak values or across selected portions of the curve. This time-adjusted segmental S/SR revealed a progressive increase in pathologically reduced segmental strain from non-obstructive disease through one-, two-, and three-vessel disease. The functional differences between one-vessel CAD and non-obstructive chest pain were minimal, with only global and segmental strain rate in early diastole (GLSR E and segmental SR E) showing statistically significant differences. Nevertheless, global strain parameters, particularly GLSR E, demonstrated good to excellent diagnostic performance for identifying high-risk patients requiring CABG. These abnormalities were most pronounced in basal segments, somewhat present in mid-ventricular segments, but notably, deviating apical segments consistently lacked post-systolic strain across all degrees of chronic CAD.

### The use of segmental strain-references

Previous studies showed significantly reduced segmental strain in chronic CAD, when focusing on territorial strain [19–21] with promising results for the detection of CAD by functional measurements in resting echocardiography. However, segmental strain comprises a challenge for achieving reliable measurements. In earlier studies [18, 22], segmental peak strains and curves were shown to deviate significantly within a normal heart, necessitating normalization at the segmental level to detect subtle pathological myocardial deformation. To our knowledge, this is the first application of time-aligned segmental strain and SR curves to measure segmental dysfunction in a chronic chest-pain population.

All deviating strain-curves exhibited consistent patterns across CAD stages, including delayed contraction/shortening, reduced systolic shortening slopes, and post-systolic strain (PSS), features previously documented in the literature [20, 21]. These patterns were predominantly observed in basal and medial segments. PSS was observed in basal septal segments of the No-CAD group as a normal phenomenon. With increasing CAD severity, the number and degree of deviating segments increased, but the curve pattern remained consistent. In regions of myocardial infarction, segments displayed initial systolic stretching, delayed contraction, and reduced shortening slopes, followed by PSS. Deviating apical strain curves rarely showed PSS, likely due to weaker contractions in neighbouring segments, as apical segments typically have higher strains than mid-ventricular segments.

Building on prior results, this study sought to improve diagnostic accuracy by identifying subtle segmental curve deviations. While previous studies suggested that resting GLS enhances CAD detection [5, 7, 11, 14, 17, 23–25], we found that although GLS decreases with increasing CAD severity, these changes remain statistically insignificant for distinguishing one-vessel CAD from non-obstructive cases. The inclusion of all CAD severities [4, 5, 7, 11, 14, 24–27] in earlier studies may have inflated detection rates for one-vessel CAD. By excluding high-grade CAD in our ROC analyses, we showed that one-vessel CAD cannot be reliably distinguished from non-obstructive cases, even with optimized normalization.

Segmental SR E [13, 20] emerged as the only significant prognosticator for PCI patients, though its diagnostic performance was limited. As in previous findings [13], only severe CAD (three-vessel disease or CABG cases) showed significantly reduced global and regional function. While prior studies have highlighted GLSR E as a reliable CAD predictor [13, 23], our results confirm its superior performance in identifying high-risk CAD or CABG indications, outperforming all other global and segmental approaches.

This study includes the largest cohort of unselected chest-pain patients with representative numbers of low-grade obstructive CAD. The limited changes observed in low-grade CAD suggest potential publication bias favouring positive findings. Smaller studies may have been underpowered to report negative results. Against expectations, GLSR E proved the most robust indicator of high-grade CAD, while GLS and GLSR S were more effective for detecting CABG patients than PCI patients or non-obstructive cases.

The inclusion of revascularized MI patients validated the segmental time-alignment methodology, demonstrating clear differences between MI and the general chest-pain population. PCI patients with low-grade CAD exhibited near-normal myocardial function, while CABG and acute MI patients showed more significant impairments. Segmental evaluation based on time-aligned segmental normal curves offers potential for improving diagnostic accuracy, especially in these high-risk cases.

### Clinical implications

Segmental definition of time-aligned normal S/SR curves appears essential to detect subtle changes in myocardial function when compared to normal hearts. This is particularly important for apical segments that often lack post-systolic strain (PSS). However, even with this robust methodology and the sensitive GLSR E measure, one-vessel CAD showed only slight functional changes, making reliable identification challenging within the chest-pain cohort.

We suggest not to assume that one-vessel CAD consistently results in clinically detectable functional reduction. Conversely, high-risk CAD can be effectively identified by reduced GLS and GLSR E values, particularly when accompanied by reductions in segmental PLS and SR E, providing high diagnostic accuracy for these advanced cases. Thus, resting speckle tracking analysis proves valuable for identifying high-grade CAD patients with high sensitivity and specificity.

The time-alignment approach demonstrated potential for utilizing strain and strain rate information beyond peak values alone. In addition, distribution patterns of pathological curves, delayed early diastolic peaks, presence of early-atrial wave fusion, and segmental double peaks in diastole represent potentially valuable criteria that could provide additional diagnostic information for detecting pathology and differentiating degrees of myocardial dysfunction.

### Limitations

This study included consecutive patients who were referred for CTCA due to chronic chest pain. Of the 485 individuals included, only 12 patients were found with two-vessel CAD, 7 patients with three-vessel CAD, and 8 patients were referred for CABG. These small groups yielded significant results with high effect sizes; however, they were underpowered to draw reliable negative conclusions. Conversely, the one-vessel CAD and PCI groups were large enough to provide reliable negative results. The chest-pain population studied was not representative of a normal group without risk factors. Previous studies have shown that hypertension, obesity, and diabetes are known contributors to reduced strain, and a healthy population with healthy hearts might have produced different reference strain values. Utilizing strain references based on standard measurements in a population without risk factors could potentially have led to different results. In this study, the SYNTAX score was not calculated, which might have resulted in a more comprehensive grouping including the impact of coronary occlusions.

## Conclusion

This study demonstrates that 1-vessel coronary artery disease cannot be reliably distinguished from non-obstructive CAD using resting speckle tracking echocardiography, GLSR E effectively identifies high-grade CAD requiring bypass grafting. Comparison with segmental S/SR references improved detection of regional dysfunction, particularly in apical segments. Time-aligned curves complement conventional global strain parameters, with pathological patterns becoming significant only in advanced coronary disease. These findings suggest resting strain analysis has greater utility for identifying high-risk patients with advanced coronary disease rather than those with mild or single-vessel disease.

## Supplementary Information

Supplementary Material 1.

Supplementary Material 2.

Supplementary Material 3.

## Data Availability

No datasets were generated or analysed during the current study.

## Abbreviation

2DSTE: Two-Dimensional Speckle Tracking Echocardiography
AF: Atrial Fibrillation
AVC: Aortic Valve Closure
BMI: Body Mass Index
CABG: Coronary Artery Bypass Grafting
CAD: Coronary Artery Disease
CAG: Coronary Angiography
CCTA: Coronary Computed Tomography Angiography
DM: Diabetes Mellitus
GLS: Global Longitudinal Strain
GLSR E: Global Longitudinal Strain Rate in Early Diastole
GLSR S: Global Longitudinal Strain Rate in Systole
HT: Hypertension
LBBB: Left Bundle Branch Block
LVEF: Left Ventricular Ejection Fraction
MI: Myocardial Infarction
PCI: Percutaneous Coronary Intervention
PSS: Post-Systolic Shortening
S/SR: Strain and Strain Rate
