# Supplementary material for: Time-aligned segmental strain as a diagnostic marker for obstructive coronary artery disease in patients with chronic chest pain
Source: Cardiovasc Ultrasound. 2026 Oct 19;24:26. doi: 10.1186/s12947-026-00388-0 (PMC13678925; doi:10.1186/s12947-026-00388-0)
Supplement: Supplementary file 1 [file 12947_2026_388_MOESM1_ESM.docx]

| Table S1: Number and percentages of segments with reduced strain values below the cut-off value of 95% normalcy | | | | | | | | | | | | | |
| --- | --- | --- | --- | --- | --- | --- | --- | --- | --- | --- | --- | --- | --- |
|  |  | Segm Strain | | Segm SR S | | Segm SR E | | Segm Strain | | Segm SR S | | Segm SR E | |
|  |  | Cut-off values below 95% segmental normalcy | | | | | | Cut-off below 95% global normalcy | | | | | |
|  | n | n | % | n | % | n | % | n | % | n | % | n | % |
| Apical | | | | | | | | | | | | | |
| No-CAD | 1721 | 83 | 4.8 | 79 | 4.6 | 85 | 4.9 | 63 | 3.7 | 77 | 4.5 | 32 | 1.9 |
| Non obstr CAD | 378 | 16 | 4.2 | 11 | 2.9 | 25 | 6.6 | 11 | 2.9 | 9 | 2.4 | 10 | 2.6 |
| 1V CAD | 406 | 19 | 4.7 | 10 | 2.5 | 28 | 6.9 | 12 | 3.0 | 16 | 3.9 | 10 | 2.5 |
| 2V CAD | 93 | 7 | 7.5 | 7 | 7.5 | 7 | 7.5 | 7 | 7.5 | 7 | 7.5 | 4 | 4.3 |
| 3V CAD | 32 | 2 | 6.3 | 0 | 0.0 | 6 | 18.8 | 2 | 6.3 | 0 | 0.0 | 2 | 6.3 |
| Revasc MI | 512 | 214 | **41.8*** | 145 | **28.3*** | 145 | **28.3*** | 193 | **37.7*** | 135 | **26.4*** | 100 | **19.5*** |
| Midt | | | | | | | | | | | | | |
| No CAD | 1740 | 85 | 4.9 | 78 | 4.5 | 86 | 4.9 | 80 | 4.6 | 89 | 5.1 | 85 | 4.9 |
| Non obstr CAD | 396 | 27 | 6.8 | 25 | 6.3 | 33 | 8.3 | 21 | 5.3 | 24 | 6.1 | 33 | 8.3 |
| 1V CAD | 407 | 28 | 6.9 | 35 | 8.6 | 39 | 9.6 | 28 | 6.9 | 36 | 8.8 | 37 | 9.1 |
| 2V CAD | 93 | 4 | 4.3 | 10 | 10.8 | 13 | 14.0 | 4 | 4.3 | 10 | 10.8 | 14 | 15.1 |
| 3V CAD | 33 | 11 | **33.3*** | 6 | **18.2*** | 11 | **33.3*** | 11 | **33.3*** | 7 | **21.2*** | 10 | **30.3*** |
| Revasc MI | 492 | 214 | **43.5*** | 162 | **32.9*** | 165 | **33.5** | 205 | **41.7*** | 166 | **33.7*** | 162 | **32.9*** |
| Basolateral | | | | | | | | | | | | | |
| No CAD | 1158 | 56 | 4.8 | 55 | 4.7 | 57 | 4.9 | 95 | 8.2 | 35 | 3.0 | 33 | 2.8 |
| Non obstr CAD | 255 | 21 | 8.2 | 19 | 7.5 | 13 | 5.1 | 28 | 11.0 | 15 | 5.9 | 11 | 4.3 |
| 1V CAD | 273 | 29 | 10.6 | 26 | 9.5* | 33 | **12.1*** | 40 | 14.7 | 20 | **7.3*** | 24 | 8.8 |
| 2V CAD | 66 | 2 | 3.0 | 6 | 9.1 | 8 | 12.1 | 5 | 7.6 | 3 | 4.5 | 5 | 7.6 |
| 3V CAD | 21 | 11 | **52.4*** | 6 | **28.6*** | 4 | 19.0 | 12 | **57.1*** | 4 | 19.0 | 1 | 4.8 |
| Revasc MI | 321 | 130 | **40.5*** | 111 | **34.6*** | 118 | **36.8*** | 149 | **46.4*** | 97 | **30.2*** | 105 | **32.7*** |
| Basoseptal | | | | | | | | | | | | | |
| No CAD | 606 | 29 | 4.8 | 27 | 4.5 | 30 | 5.0 | 48 | 7.9 | 44 | 7.3 | 97 | 16.0 |
| Non obstr CAD | 136 | 8 | 5.9 | 10 | 7.4 | 7 | 5.1 | 16 | 11.8 | 10 | 7.4 | 28 | 20.6 |
| 1V CAD | 141 | 6 | 4.3 | 4 | 2.8 | 10 | 7.1 | 12 | 8.5 | 10 | 7.1 | 26 | 18.4 |
| 2V CAD | 32 | 1 | 3.1 | 2 | 6.3 | 1 | 3.1 | 2 | 6.3 | 3 | 9.4 | 8 | 25.0 |
| 3V CAD | 13 | 3 | 23.1 | 2 | 15.4 | 5 | 38.5* | 4 | 30.8 | 3 | 23.1 | 8 | **61.5*** |
| Revasc MI | 165 | 65 | **39.4*** | 44 | **26.7*** | 45 | **27.3*** | 69 | **41.8*** | 47 | **28.5*** | 86 | **52.1*** |
|  |  |  |  |  |  |  |  |  |  |  |  |  |  |
| All Segments | | | | | | | | | | | | | |
| No CAD | 5525 | 253 | 4.8 | 239 | 4.6 | 258 | 4.9 | 286 | 5.5 | 245 | 4.7 | 247 | 4.7 |
| Non obstr CAD | 1165 | 72 | 6.2 | 65 | 5.6 | 78 | 6.7 | 76 | 6.5 | 58 | 5.0 | 82 | 7.0 |
| 1V CAD | 1225 | 82 | 6.7 | 75 | 6.1 | 110 | **9.0*** | 92 | 7.5 | 82 | 6.7 | 97 | **7.9*** |
| 2V CAD | 283 | 14 | 4.9 | 25 | 8.8 | 29 | **10.2*** | 18 | 6.3 | 23 | 8.1 | 31 | **10.9*** |
| 3V CAD | 99 | 27 | **27.3*** | 14 | **14.1*** | 26 | **26.3*** | 29 | **29.3*** | 14 | **14.1*** | 21 | **21.2*** |
| Revasc MI | 1490 | 623 | **41.8*** | 462 | **31.0*** | 473 | **31.7*** | 616 | **41.3*** | 445 | **29.9*** | 453 | **30.4*** |
| P<0.05 for the comparison towards the no CAD group. Segm: segmental; SR: strain rate; 1,2,3 V CAD: 1,2,3-Vessel coronary artery disease; revasc MI: revascularized myocardial infarction; obstr: obstructive | | | | | | | | | | | | | |

| Table S2: ROC curve analysis between No-CAD and non-obstructive CAD against acute revascularized infarctions | | | | |
| --- | --- | --- | --- | --- |
|  | Area | p-value | CI  lower bound | CI  upper bound |
| Segm Curve dev strain systole (%) | 0.782 | <0.0001 | 0.725 | 0.839 |
| Segm Curve dev E-wave (s^-1^) | 0.764 | <0.0001 | 0.706 | 0.823 |
| Segm PL Strain dev (%) | 0.781 | <0.0001 | 0.724 | 0.839 |
| Segm PL SR S dev (s^-1^) | 0.680 | <0.0001 | 0.613 | 0.748 |
| Segm PL SR E dev (s^-1^) | 0.782 | <0.0001 | 0.729 | 0.834 |
| GLS (%) | 0.750 | <0.0001 | 0.692 | 0.807 |
| GL SR S (s^-1^) | 0.645 | <0.0001 | 0.576 | 0.714 |
| GL SR E (s^-1^) | 0.605 | <0.0001 | 0.542 | 0.669 |
| Segm: segmental; GLS: global longitudinal strain; PL: peak longitudinal; GL SR S: Global longitudinal strain rate in systole; GL SR E: global longitudinal SR early diastole; CI: confidence interval | | | | |

Figure S1: Average standardized segmental strain curves within the No-CAD group, stratified by percentile ranges of deviation from the segmental reference curve. Curves represent segments within the 10–25th, 5–10th, and <5th percentile of deviation, calculated over the systolic phase

Figure S2. Average standardized segmental strain rate curves within the No-CAD group, stratified by percentile ranges of deviation from the segmental reference curve. Curves represent segments within the 10–25th, 5–10th, and <5th percentile of deviation, calculated over the first third of diastole.
